# The Report of the War Office Committee on "Shell-Shock"

**Published:** 1922-10

**Authors:** 


### THE REPORT OF THE WAR OFFICE COMMITTEE ON "SHELL-SHOCK."

IN August, 1920, the Army Council appointed a committee to consider the different types of hysteria and traumatic neurosis, commonly called "shell-shock," of which the war has furnished great experience. The committee consisted largely of military and naval medical officers who themselves had considerable experience of "shell-shock" in the war, and included eminent neurologists. They heard evidence from a number of distinguished men, representing opinion on the military, medical and legal aspect of the subject, and also from officers and men who had suffered from "shell-shock." They have made a lengthy and exhaustive report which must long remain the authoritative work on the subject.

The Committee declare the term "shell-shock" to be a gross and costly misnomer which should be abolished. The war produced no new nervous disorders, but, owing to special conditions, the disorders produced appeared in some cases in an aggravated form. They are divisible into three main classes: (1) Genuine concussion, without visible wound, as a result of shell explosion. Such cases were relatively few. (2) Emotional shock, either acute in men with a neurotic disposition, or developing slowly as a result of prolonged strain and terrifying experience, the final breakdown being sometimes brought about by some relatively trivial cause. (3) Nervous and mental exhaustion, the result of prolonged strain and hardship. In many cases the three factors of commotional and emotional shock and exhaustion were combined.

Any type of person may suffer from one or other form of neurosis if exposed for sufficient time to the conditions of modern warfare. It is difficult to say beforehand what type of man is most likely to break down. But certain individuals are unlikely ever to become efficient fighting soldiers. Much can be done by training. The more gradual the training, the better the staying power. In prevention the inculcation of morale and discipline stand first. Included under morale are pride of regiment, belief in the cause, mutual confidence between officers and men, and the feeling that a man is part of a corporate whole. Physical comfort, so far as circumstances allow, adequate rest and recreation are important adjuncts. But no human being, however constituted or trained, can resist the direct effect of the bursting of high-explosive shells. A large proportion of soldiers who were blown up or came into the class of commotional shock showed exactly the same symptoms as those suffering from the emotional form. There was no evidence of organic injury of the nervous system, and the same causes which are at the root of the emotional form were found. There was, for instance, a family history of tuberculosis or of nervous disease, or a personal history of previous "shell-shock" or of neurosis.

Emotional "shell-shock" is a term used to imply that the exciting cause is emotional. Though common on the battlefield, it may be acquired elsewhere. It was common on home service, and in its hysterical form especially often developed after weeks of absence from the scenes of fighting. It formed about 80 per cent. of all cases and was brought about by a variety and combination of causes. There was first some kind of mental or physical exhaustion of a cumulative nature. Then some emotional disturbance acted as an exciting cause. The conditions determining exhaustion included noise, loss of sleep, fatigue, discomfort, insufficient food, immoderate use of alcohol, infectious diseases, the pain of wounds and sores, poison gases. All soldiers have fear, but this state is generally controlled. In the large majority of cases of emotional "shell-shock" there was in the family or personal history evidence of weakness or instability of the nervous system.

With regard to commotional "shell-shock," the bursting of a shell may cause commotion of the nervous system by direct aerial percussion. Most of the witnesses stated that they had seen men lying dead from a shell explosion without any visible injury, but few of them could give evidence that proved death to have been caused by aerial percussion. In such cases no changes visible to the naked eye were found in the brain, but microscopic examination revealed ruptures of small vessels with hæmorrhages.

The question arose whether "shell-shock" should rank as a battle casualty. The Committee make the following recommendations: Concussion or commotion attended by loss of consciousness and evidence of organic lesion of the central nervous system should be classified as a battle casualty. (2) No case of psycho-neurosis or mental breakdown, even when attributable to a shell explosion, should be classified as a battle casualty, any more than sickness or disease is so regarded. (3) In all doubtful cases it is desirable to have the classification determined by a Board of expert medical officers after observation of the patient in a neurological hospital.

The distinction between cowardice and "shell-shock" is of great importance, as cowardice is a military crime for which the death penalty may be exacted. Cowardice may be regarded as lack of or failure to show requisite courage, but after much stress the brave may temporarily fail to show their wonted courage without deserving the opprobious term. Fear is the chief factor in both cowardice and emotional "shell-shock"; it is an emotion common to all, and very brave men frankly acknowledged it. Obviously fear alone does not constitute cowardice. Prof. G. Roussy, a French expert, who gave evidence, noted the difficulty of distinguishing between cowardice and emotional "shell-shock." He defined cowardice as "lack of self-control in the presence of danger." But an English expert, Dr. E. Mapother, was "not prepared to make a decision between cowardice and 'shell-shock.'" "Shell-shock" he regarded as "persistent

(*Continued on page 402.*)

### REPORT ON "SHELL-SHOCK."

(*Continued from page 393.*)

and chronic fear." Another expert, Dr. W. Johnson, thought that when symptoms of fear tremors, sweating and rapid action of the heart persisted or revived on slight emotional stimulation, neurosis was present. As regards expert medical advice and evidence in court-martial cases, the system pursued in the war seems to have been satisfactory. When any medical question or doubt arose before or at a trial or on subsequent review of the proceedings, the best possible expert advice available was placed at the disposal of the authorities. The Committee conclude that : (1) The military aspect of cowardice is justified. (2) Seeming cowardice may be beyond the individual control. (3) Experienced and specialised medical opinion is required to decide in possible cases of war neurosis of doubtful character. (4) A man who has proved his courage should receive special consideration in case of subsequent lapse.

